# Supplementary material for: Equatorial waves as useful precursors to tropical cyclone occurrence and intensification
Source: Nat Commun. 2023 Jan 31;14:511. doi: 10.1038/s41467-023-36055-5 (PMC9889306; doi:10.1038/s41467-023-36055-5)
Supplement: Supplementary file 1 — Supplementary Information [file 41467_2023_36055_MOESM1_ESM.pdf]

Supplementary Information

Equatorial waves as useful precursors to tropical cyclone occurrence and intensification

Xiangbo Feng[1*], Gui-Ying Yang[1], Kevin I. Hodges[1], John Methven[2]

[1] National Centre for Atmospheric Science and Department of Meteorology, University of Reading, Reading, United Kingdom

[2] Department of Meteorology, University of Reading, Reading, United Kingdom

* Corresponding author: xiangbo.feng@reading.ac.uk

Supplementary Table 1: Percentages of global pre-tropical cyclogenesis (pre-TCG) events matched in-phase to westward-moving equatorial waves

Percentages of all pre-TCG events, and pre-TCG events with strong vorticity, matched in-phase to westward-moving equatorial waves in the globe, Northern Hemisphere (NH) and Southern Hemisphere (SH), over 1980–2018. Westward-moving equatorial waves include Westward-moving Mixed Rossby-Gravity wave (WMRG), mode number 1 Rossby wave (R1) and mode number 2 Rossby wave (R2). Intensity of pre-TC vortex is defined by the absolute value of relative vorticity at the pre-TC vortex centre (in the units of Cyclonic Vorticity Unit, CVU, 1 CVU=$1.0*10^{-5}$ s^{-1}); pre-TCG events with strong vorticity are the events when the pre-TCG vorticity > 3 CVU. In-phase matching includes: (i) matched to at least one type (AL-1wave), matched to at least two types (AL-2wave), and matched to all three types of westward-moving waves (3wave), and (ii) matched to WMRG, R1 and R2 waves, respectively.

	AL-1wave (%)	AL-2wave (%)	3wave (%)	WMRG (%)	R1 (%)	R2 (%)
pre-TCG in globe	64	29	8	35	36	30
pre-TCG in NH	66	31	9	36	37	32
pre-TCG in SH	59	24	6	30	32	26
strong pre-TCG in globe	73	38	15	43	43	39
strong pre-TCG in NH	76	43	18	46	47	43
strong pre-TCG in SH	64	28	8	36	35	29

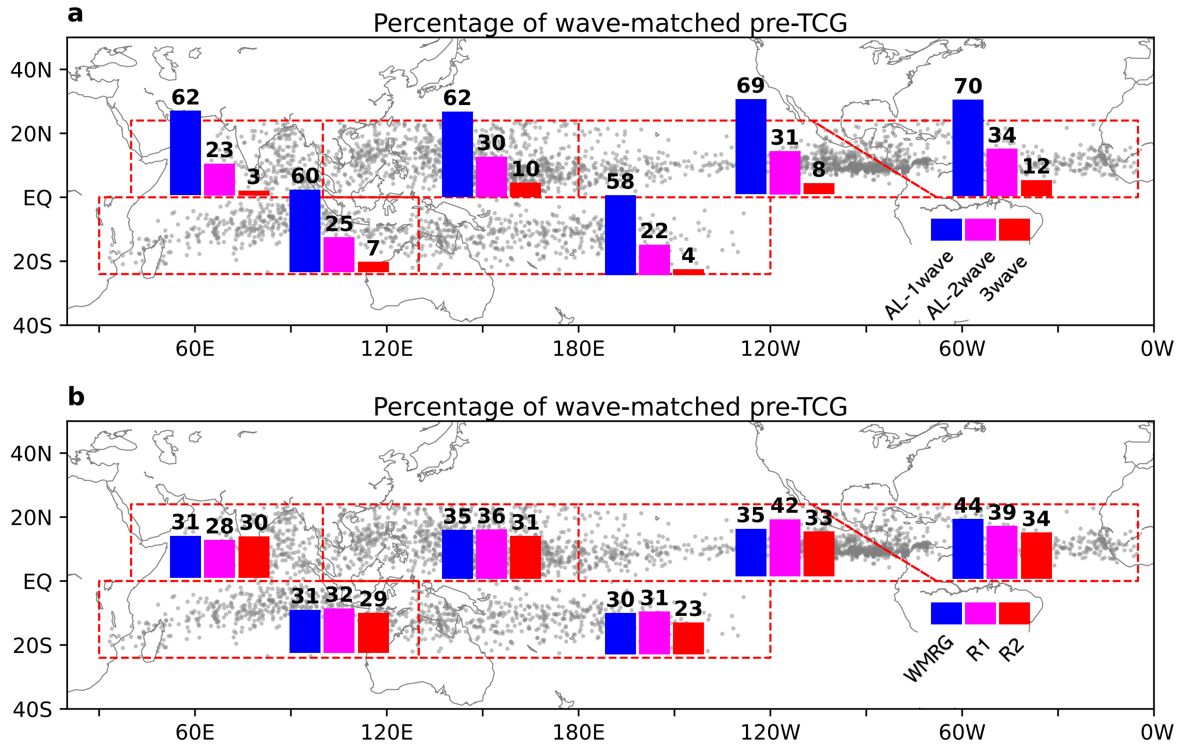

Supplementary Figure 1: Percentages of all pre-tropical cyclogenesis (pre-TCG) events matched in-phase to equatorial waves

(a) Percentages of all pre-TCG events matched in-phase to at least one type (AL-1wave, blue bar), at least two types (AL-2wave, magenta bar), and all three types of westward-moving waves (3wave, red bar), for each ocean basin, over 1980–2018.

(b) as (a), but for percentages of all pre-TCG events matched in-phase to each type of westward-moving waves: Westward-moving Mixed Rossby-Gravity wave (WMRG, blue bar), mode number 1 Rossby wave (R1, magenta bar), and mode number 2 Rossby wave (R2, red bar). Grey dots show the pre-TCG position in 24°N–24°S; red dashed lines show the boundaries of each ocean basin.

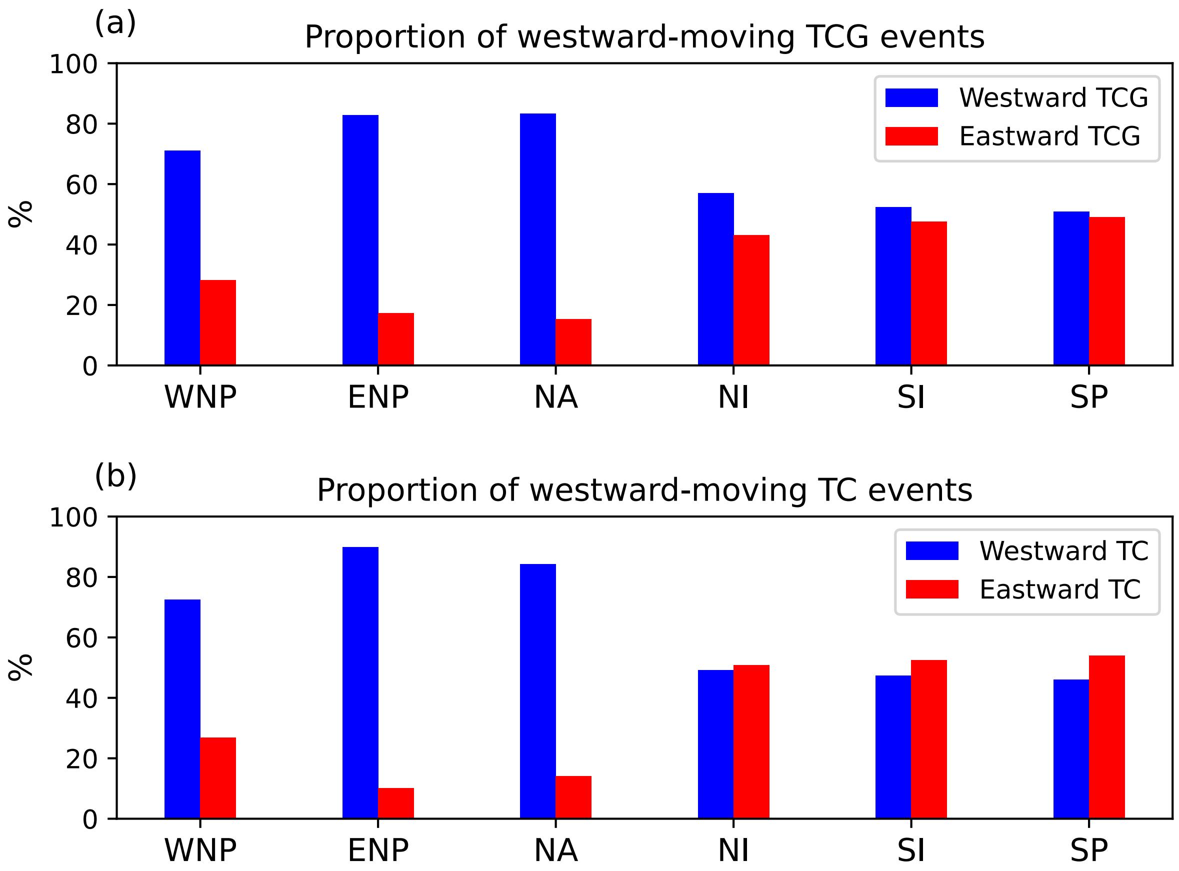

Supplementary Figure 2: Proportion of westward-moving tropical cyclones (TCs) in each basin

(a) Percentages of pre-tropical cyclogenesis (pre-TCG) events with westward-moving (blue bar) or eastward-moving (red bar) vortex in each ocean basin, over 1980–2018. (b) as (a), but for percentages of westward-moving (blue bar) or eastward-moving (red bar) TC. For a pre-TCG event with westward-moving vortex, the 2nd track point is further west than the 1st track point; for a pre-TCG event with eastward-moving vortex, the 2nd track point is further east than the 1st track point. Westward-moving TCs have the following criterion: the poleward-most track point, or the track point closest to 24°N and 24°S, whenever it reaches first, is further west than the vortex centre of pre-TCG by at least 5°. In contrast, the eastward-moving TCs are the storms that do not meet above criterion.

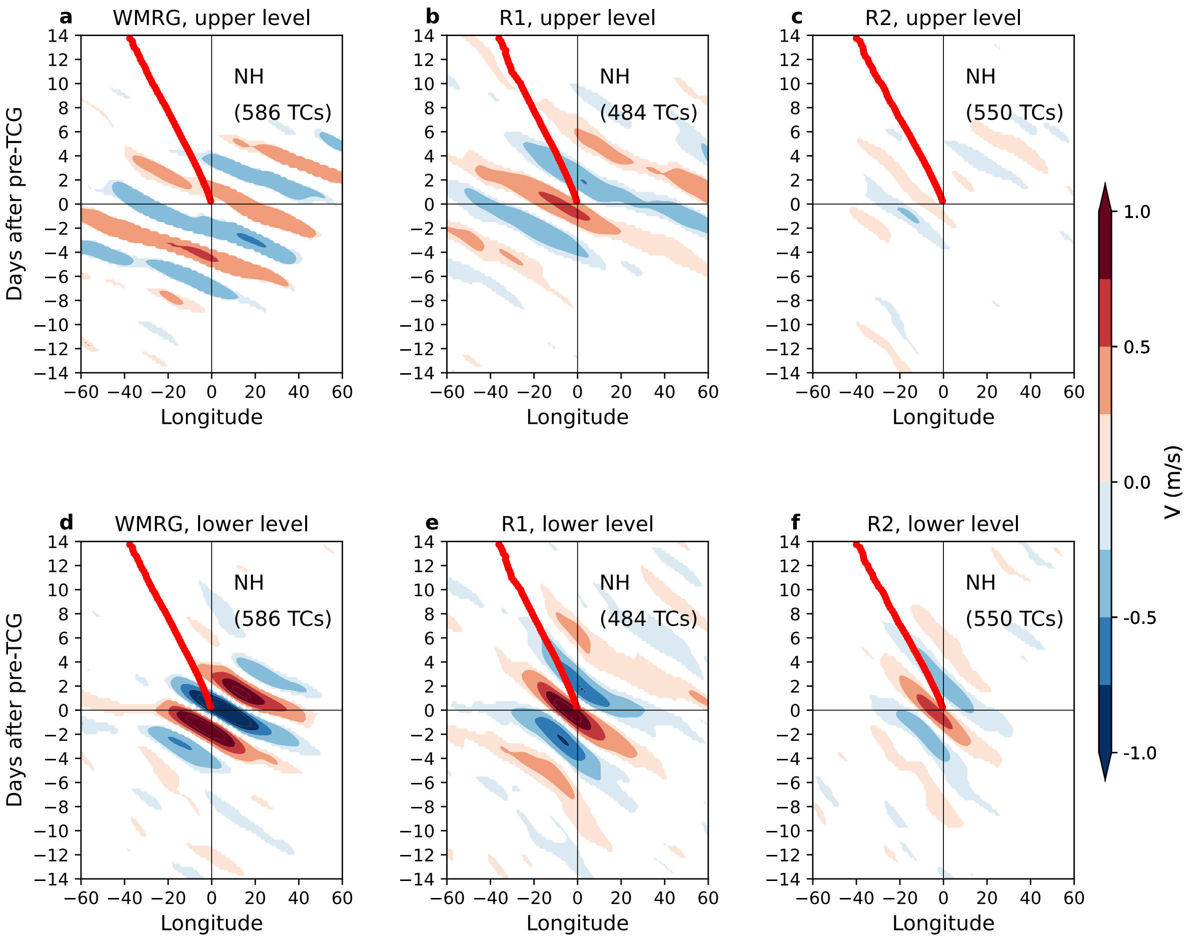

Supplementary Figure 3: Hovmöller diagram of equatorial waves when they are matched out-of-phase to pre-tropical cyclogenesis (pre-TCG) events in the Northern Hemisphere (NH)

(a) Hovmöller diagram of composite means (shading) of Westward-moving Mixed Rossby-Gravity wave (WMRG) meridional winds (*V*) at the equator (0°N) in the upper troposphere onto pre-TCG events that are matched out-of-phase to the waves, with respect to the vortex longitude and event time of pre-TCG, in the NH. Only composite significant at the 95% confidence level is shown. The total number of pre-TCG events matched out-of-phase to each type of wave is provided in the top right; thick red line shows the averaged longitude of storm track, with respect to the vortex longitude and event time of pre-TCG.

(b, c) as (a), but for mode number 1 Rossby wave (R1) and mode number 2 Rossby wave (R2) meridional winds at 8°N and 13°N, respectively. 0°N, 8°N and 13°N are chosen because WMRG, R1 and R2 waves have the maximum values of *V* at these three latitudes, respectively. (d-f) as (a-c), but for the lower troposphere. The upper troposphere is equally averaged over 100, 150, 200, 250 and 300 hPa, while the lower troposphere is averaged over 900, 850, 800, 750 and 700 hPa.

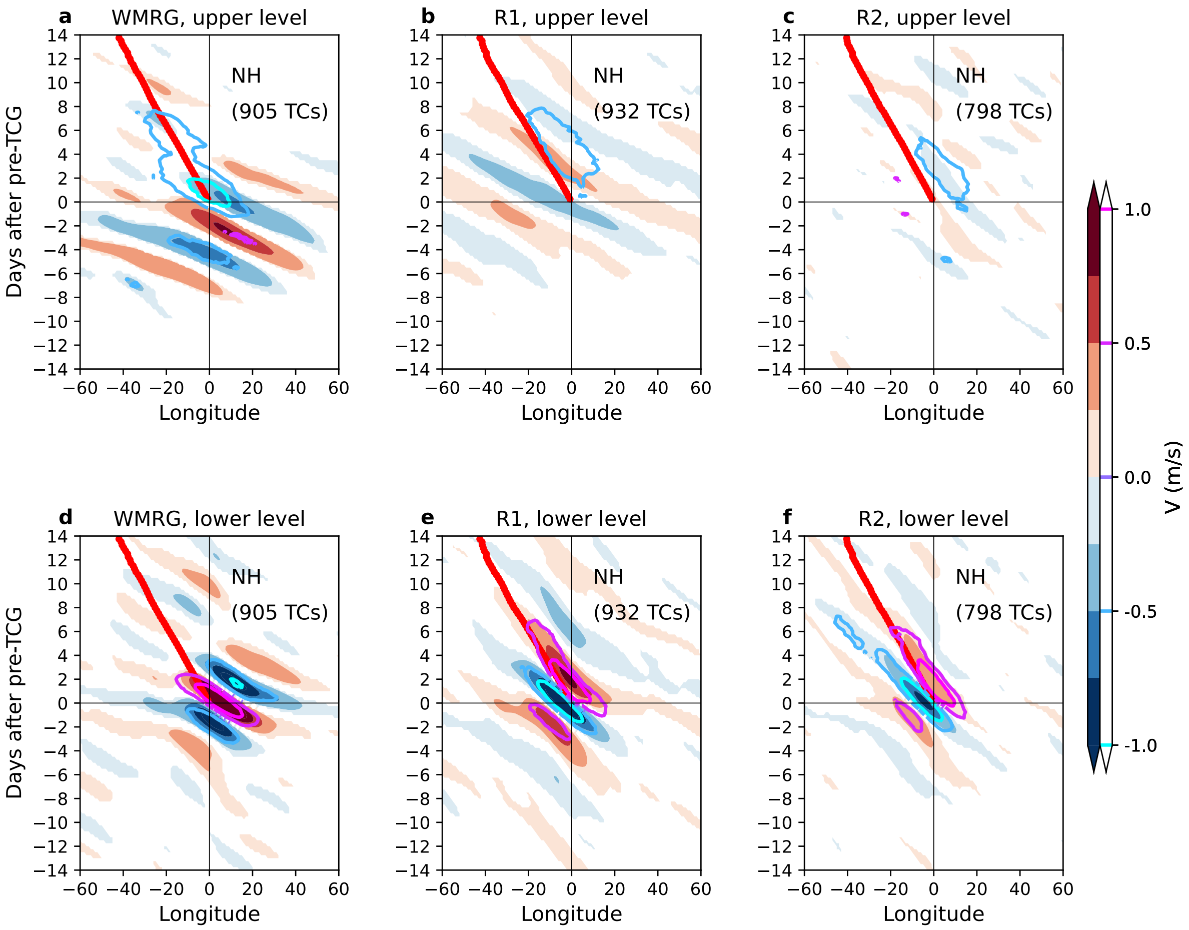

Supplementary Figure 4: Hovmöller diagram of equatorial waves and unfiltered wind anomalies when waves are matched in-phase to pre-tropical cyclogenesis (pre-TCG) events in the Northern Hemisphere (NH)

(a) Hovmöller diagram of composite means (shading) of Westward-moving Mixed Rossby-Gravity wave (WMRG) meridional winds (V) at the equator (0°N) in the upper troposphere onto pre-TCG events that are matched in-phase to the waves, with respect to the vortex longitude and event time of pre-TCG, in the NH. Contour lines are composite means of unfiltered meridional wind anomalies at the equator (0°N) in the upper troposphere onto pre-TCG events that are matched in-phase to WMRG waves. Only composite significant at the 95% confidence level is shown. The total number of pre-TCG events matched in-phase to each type of wave is provided in the top right; thick red line shows the averaged longitude of storm track, with respect to the vortex longitude and event time of pre-TCG.

(b, c) as (a), but for mode number 1 Rossby wave (R1) and mode number 2 Rossby wave (R2) meridional winds (shading), and unfiltered meridional wind anomalies (contour) at 8°N and 13°N, respectively. 0°N, 8°N and 13°N are chosen because WMRG, R1 and R2 waves have the maximum values of V at these three latitudes, respectively. (d-f) as (a-c), but for the lower troposphere. The upper troposphere is equally averaged over 100, 150, 200, 250 and 300 hPa, while the lower troposphere is averaged over 900, 850, 800, 750 and 700 hPa.

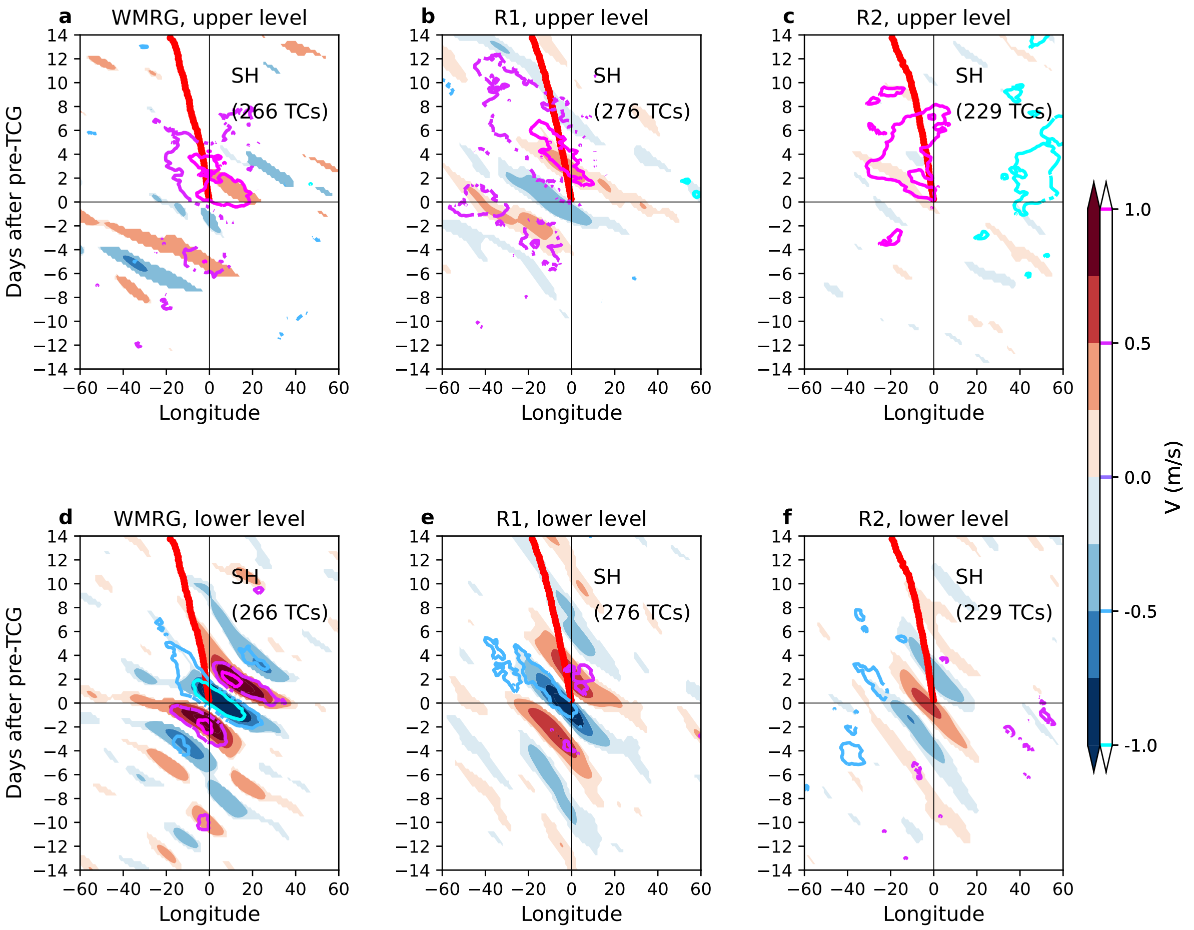

Supplementary Figure 5: Hovmöller diagram of equatorial waves and unfiltered wind anomalies when waves are matched in-phase to pre-tropical cyclogenesis (pre-TCG) events in the Southern Hemisphere (SH)

(a) Hovmöller diagram of composite means (shading) of Westward-moving Mixed Rossby-Gravity wave (WMRG) meridional winds (*V*) at the equator (0°N) in the upper troposphere onto pre-TCG events that are matched in-phase to the waves, with respect to the vortex longitude and event time of pre-TCG, in the SH. Contour lines are composite means of unfiltered meridional wind anomalies at the equator (0°N) in the upper troposphere onto pre-TCG events that are matched in-phase to WMRG waves. Only composite significant at the 95% confidence level is shown. The total number of pre-TCG events matched in-phase to each type of wave is provided in the top right; thick red line shows the averaged longitude of storm track, with respect to the vortex longitude and event time of pre-TCG.

(b, c) as (a), but for mode number 1 Rossby wave (R1) and mode number 2 Rossby wave (R2) meridional winds (shading), and unfiltered meridional wind anomalies (contour) at 8°S and 13°S, respectively. 0°N, 8°S and 13°S are chosen because WMRG, R1 and R2 waves have the maximum values of *V* at these three latitudes, respectively. (d-f) as (a-c), but for the lower troposphere. The upper troposphere is equally averaged over 100, 150, 200, 250 and 300 hPa, while the lower troposphere is averaged over 900, 850, 800, 750 and 700 hPa.

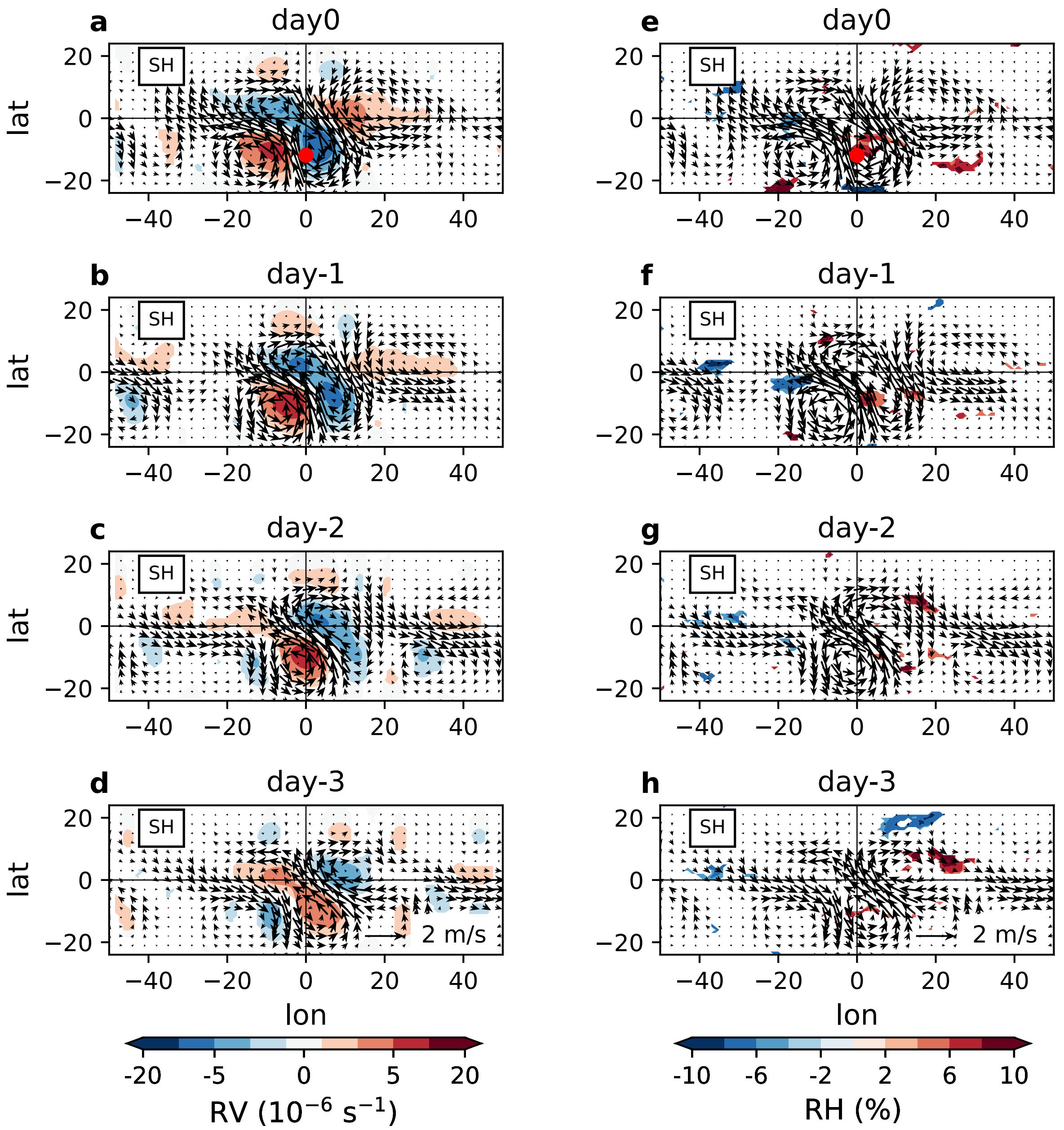

Supplementary Figure 6: Co-existing and pre-existing equatorial waves related to pre-tropical cyclogenesis (pre-TCG) events in the Southern Hemisphere (SH)

(a) Composite means of wave horizontal winds (vectors) and relative vorticity (RV, in the units of 10^{-6} s^{-1}; shading) in the lower troposphere onto pre-TCG events that are simultaneously matched in-phase to Westward-moving Mixed Rossby-Gravity wave (WMRG), mode number 1 Rossby wave (R1) and mode number 2 Rossby wave (R2), with respect to the vortex longitude and event time of pre-TCG, in the SH. Only composite of relative vorticity of wave winds significant at the 95% confidence level is shown. Winds are combined from WMRG, R1 and R2 wave winds. Red dot shows the averaged latitude of pre-TCG vortex.

(b-d) as (a), but for composite means 1–3 days before pre-TCG events. (e-h) as (a-d), but with shading showing composite means of relative humidity anomaly at 700hPa (RH, in the units of %). The lower troposphere is equally averaged over 900, 850, 800, 750 and 700 hPa.

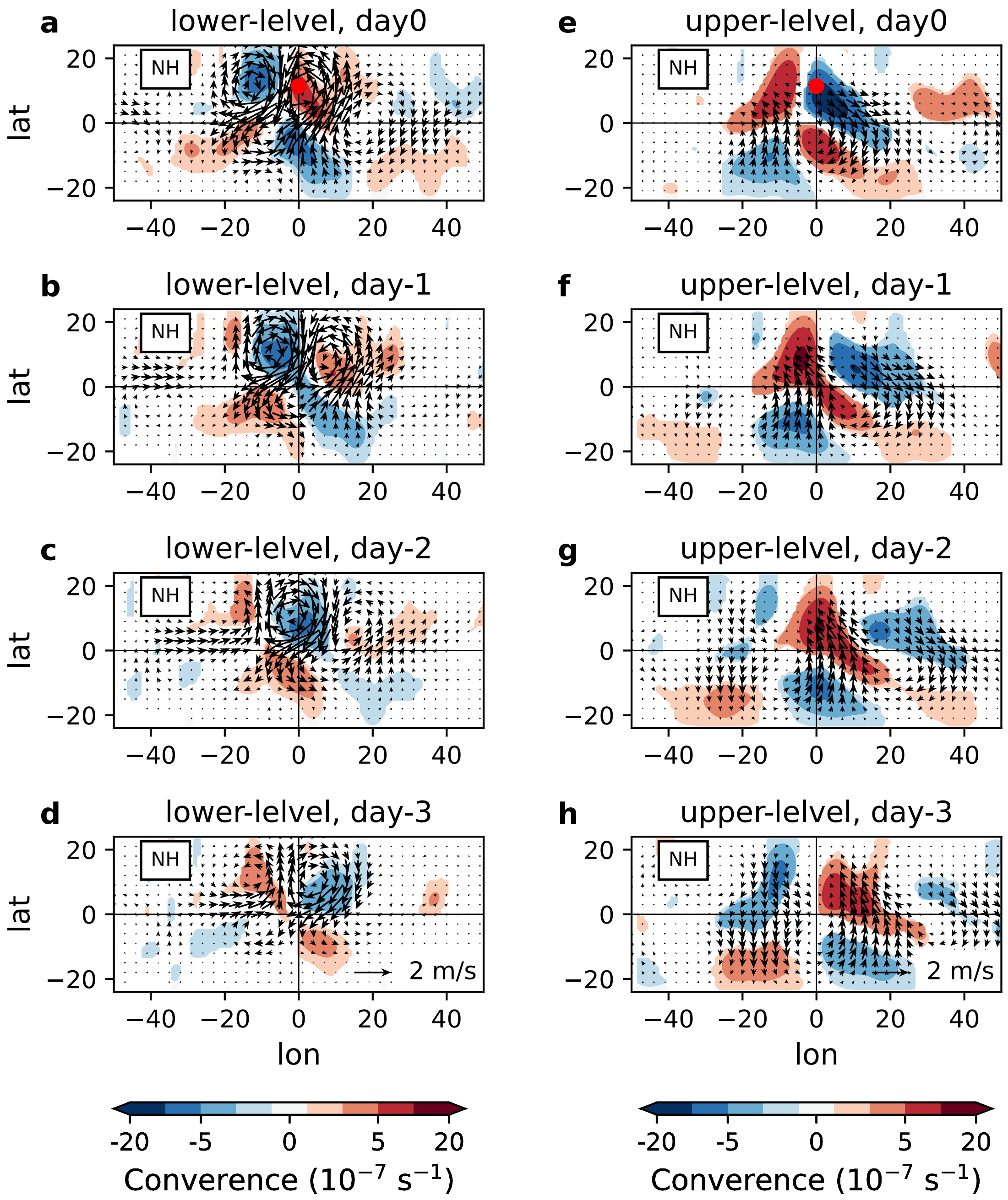

Supplementary Figure 7: Co-existing and pre-existing equatorial waves related to pre-tropical cyclogenesis (pre-TCG) events in the Northern Hemisphere (NH)

(a) Composite means of wave horizontal winds (vectors) and wave convergence (shading, in the units of 10^{-6} s^{-1}) in the lower troposphere onto pre-TCG events that are simultaneously matched in-phase to Westward-moving Mixed Rossby-Gravity wave (WMRG), mode number 1 Rossby wave (R1) and mode number 2 Rossby wave (R2), with respect to the vortex longitude and event time of pre-TCG, in the NH. Only composite of wave convergence significant at the 95% confidence level is shown. Winds are combined from WMRG, R1 and R2 wave winds. Red dot shows the averaged latitude of pre-TCG vortex. (b-d) as (a), but for composite means 1–3 days before pre-TCG events. (e-h) as (a-d), but for wave horizontal winds and wave convergence in the upper troposphere. The upper troposphere is equally averaged over 100, 150, 200, 250 and 300 hPa, while the lower troposphere is averaged over 900, 850, 800, 750 and 700 hPa.

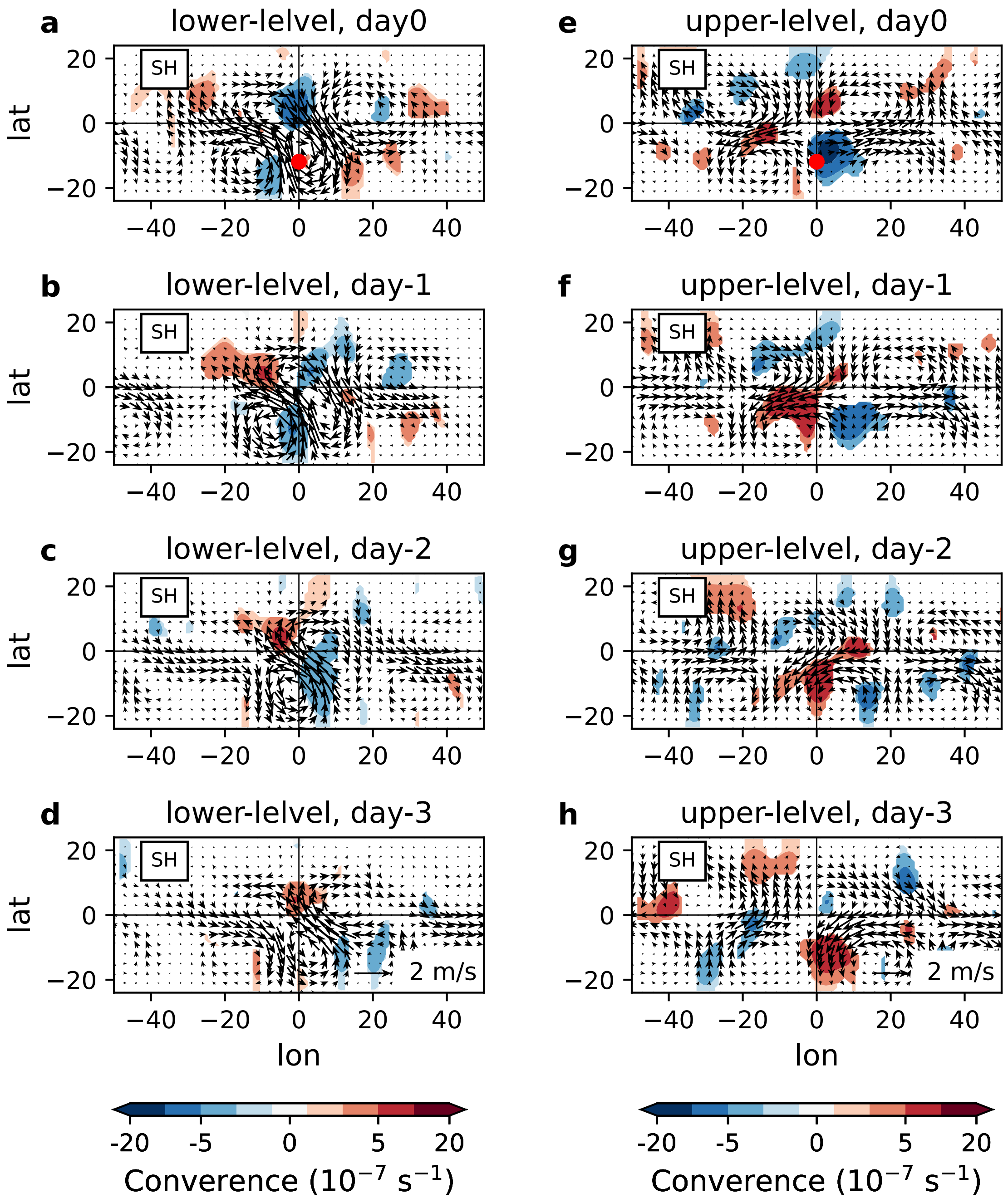

Supplementary Figure 8: Co-existing and pre-existing equatorial waves related to pre-tropical cyclogenesis (pre-TCG) events in the Southern Hemisphere (SH)

(a) Composite means of wave horizontal winds (vectors) and wave convergence (shading, in the units of 10^{-6} s^{-1}) in the lower troposphere onto pre-TCG events that are simultaneously matched in-phase to Westward-moving Mixed Rossby-Gravity wave (WMRG), mode number 1 Rossby wave (R1) and mode number 2 Rossby wave (R2), with respect to the vortex longitude and event time of pre-TCG, in the SH. Only composite of wave convergence significant at the 95% confidence level is shown. Winds are combined from WMRG, R1 and R2 wave winds. Red dot shows the averaged latitude of pre-TCG vortex.

(b-d) as (a), but for composite means 1–3 days before pre-TCG events. (e-h) as (a-d), but for wave horizontal winds and wave convergence in the upper troposphere. The upper troposphere is equally averaged over 100, 150, 200, 250 and 300 hPa, while the lower troposphere is averaged over 900, 850, 800, 750 and 700 hPa.

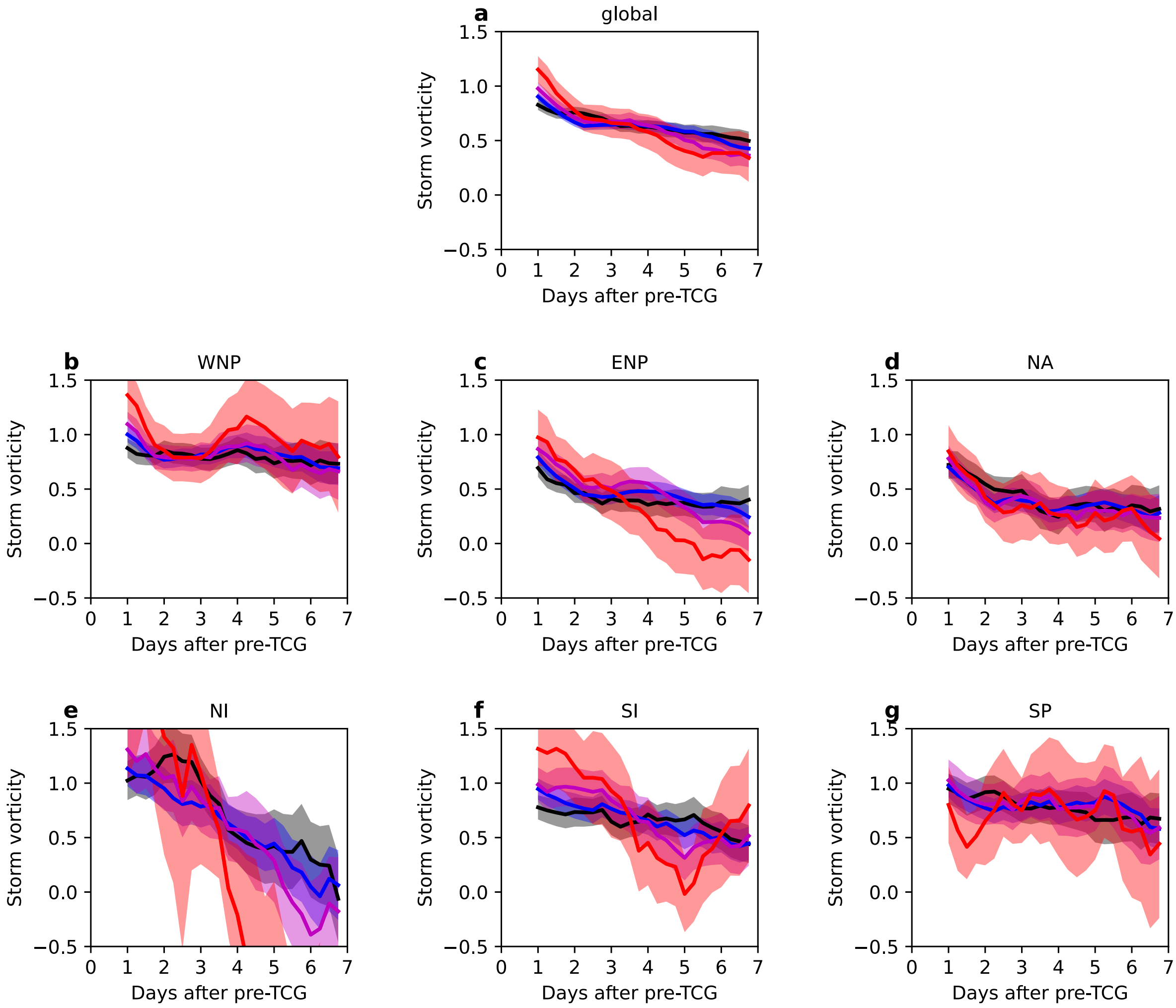

Supplementary Figure 9: Global and basin-wide pre-tropical cyclone (pre-TC) intensification rate related to equatorial waves

(a) Global pre-TC 24-hour intensification rate conditional on the phase matching between pre-TCG events and equatorial waves, as a function of the time after pre-TCG events. The phase matching includes: not matched in-phase to any type of westward-moving waves (black line), matched in phase to at least one type (blue line), matched in-phase to at least two types (magenta line), and matched in-phase to all three types of westward-moving waves (red line). The shading shows the 95% confidence interval of the mean. Pre-TC intensity is defined by the absolute value of relative vorticity of the pre-TC vortex (in the units of Cyclonic Vorticity Unit, CVU, 1 CVU=$1.0*10^{-5}$ s^{-1}). The minimum sample size for each 6-hourly time interval is 10 storms. Westward-moving waves include Westward-moving Mixed Rossby-Gravity wave (WMRG), mode number 1 Rossby wave (R1) and mode number 2 Rossby wave (R2).

(b-g) as (a), but for pre-TCs in each ocean basin. WNP =Western North Pacific, ENP=Eastern North Pacific, NA =North Atlantic, NI =North Indian Ocean, SI =South Indian Ocean and SP =South Pacific (ocean basins are defined in Figure 1).

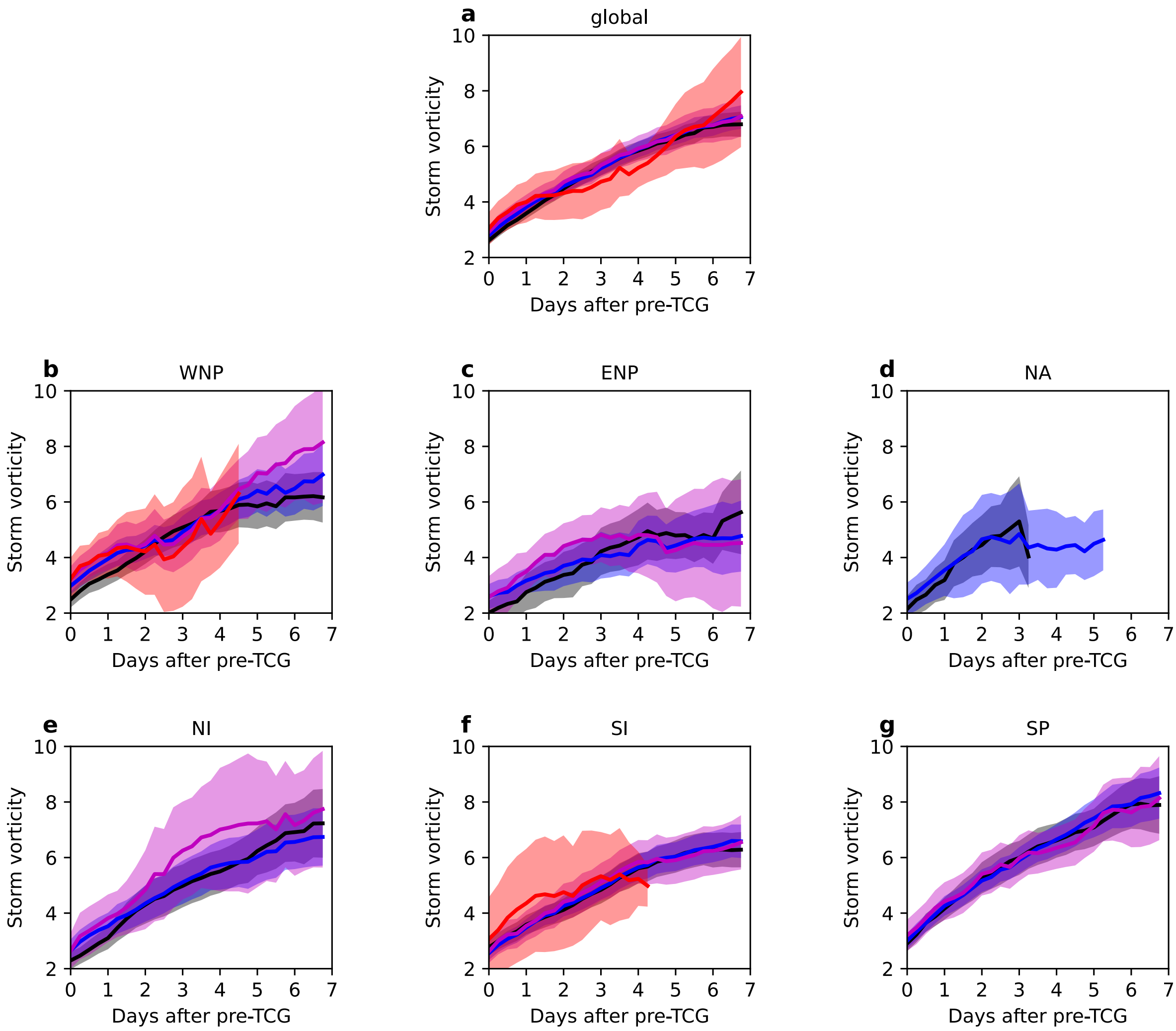

Supplementary Figure 10: Global and basin-wide eastward-moving pre-tropical cyclone (pre-TC) intensity related to equatorial waves

(a) Global eastward-moving pre-TC intensity conditional on the phase matching between pre-tropical cyclogenesis (pre-TCG) events and equatorial waves, as a function of the time after pre-TCG events. Eastward-moving TCs are those storms that travel to the east during their time in the tropical band (24°N–24°S). The phase matching includes: not matched in-phase to any type of westward-moving waves (black line), matched in phase to at least one type (blue line), matched in-phase to at least two types (magenta line), and matched in-phase to all three types of westward-moving waves (red line). The shading shows the 95% confidence interval of the mean. Pre-TC intensity is defined by the absolute value of relative vorticity of the pre-TC vortex (in the units of Cyclonic Vorticity Unit, CVU, 1 CVU=1.0*10^{-5} s^{-1}). The minimum sample size for each 6-hourly time interval is 10 storms. Westward-moving waves include Westward-moving Mixed Rossby-Gravity wave (WMRG), mode number 1 Rossby wave (R1) and mode number 2 Rossby wave (R2).

(b-g) as (a), but for pre-TCs in each ocean basin. WNP =Western North Pacific, ENP=Eastern North Pacific, NA =North Atlantic, NI =North Indian Ocean, SI =South Indian Ocean and SP =South Pacific (ocean basins are defined in Figure 1).

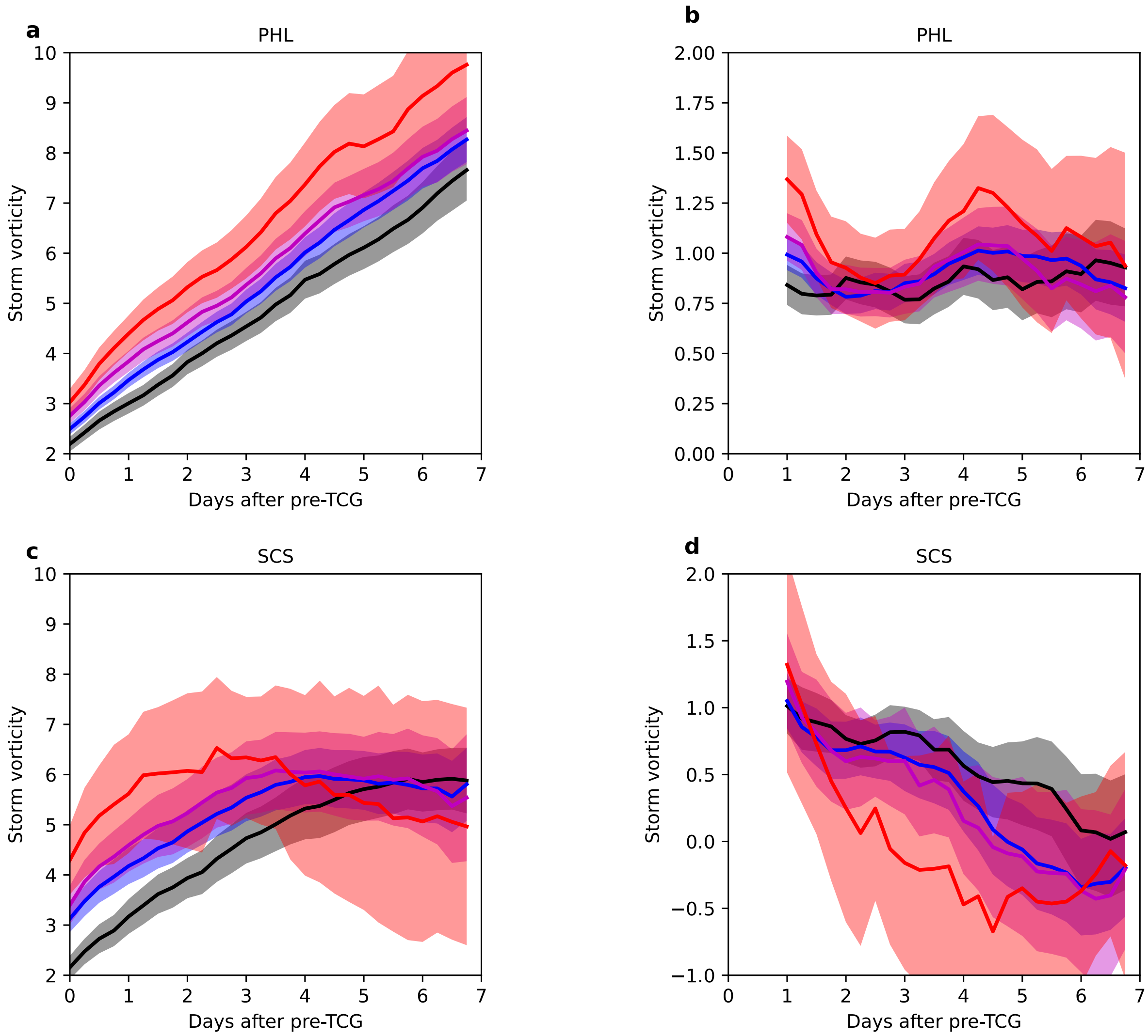

Supplementary Figure 11: Pre-tropical cyclone (pre-TC) intensity and intensification rate related to equatorial waves in the two regions of the western North Pacific

(a) Intensity of pre-TCs formed east of the Philippines (PHL; 120-180°E, 0-24°N) conditional on the phase matching between pre-tropical cyclogenesis (pre-TCG) events and equatorial waves, as a function of the time after pre-TCG events. The phase matching includes: not matched in-phase to any type of westward-moving waves (black line), matched in phase to at least one type (blue line), matched in-phase to at least two types (magenta line), and matched in-phase to all three types of westward-moving waves (red line). The shading shows the 95% confidence interval of the mean. Pre-TC intensity is defined by the absolute value of relative vorticity of the pre-TC vortex (in the units of Cyclonic Vorticity Unit, CVU, 1 CVU=1.0*10^{-5} s^{-1}). The minimum sample size for each 6-hourly time interval is 10 storms. Westward-moving waves include Westward-moving Mixed Rossby-Gravity wave (WMRG), mode number 1 Rossby wave (R1) and mode number 2 Rossby wave (R2).

(b) as (a), but for 24-hour intensification rate of pre-TCs. (c, d) as (a, b) but for pre-TCs formed in the South China Sea (SCS; 100-120°E, 0-24°N).

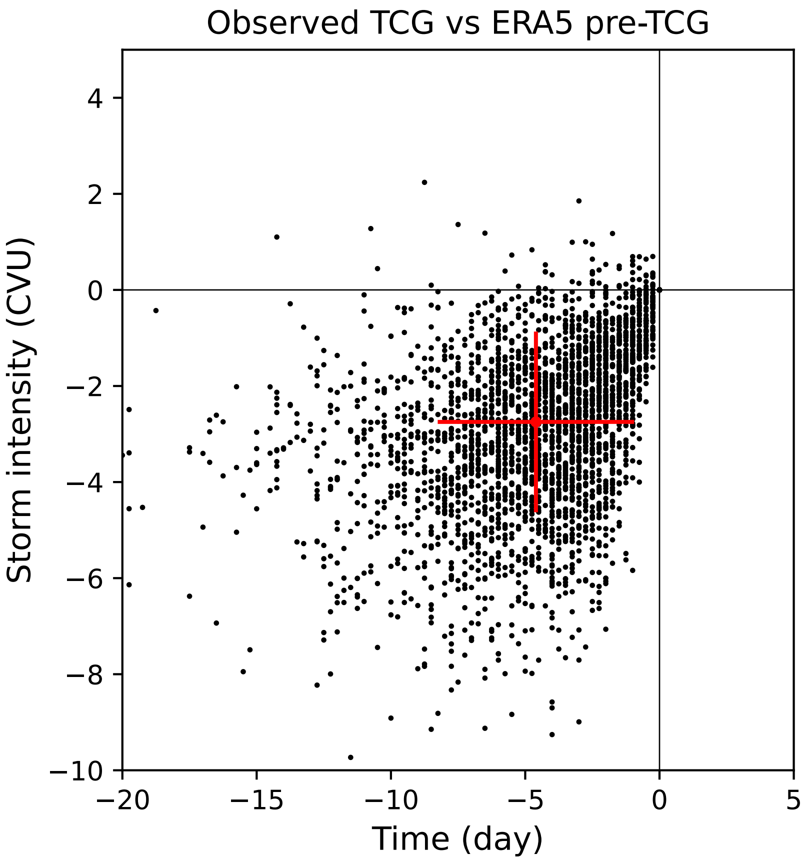

Supplementary Figure 12: Earlier stage of ERA5 pre-tropical cyclogenesis (pre-TCG) compared to observed tropical cyclogenesis (TCG)

Differences in the time and intensity (i.e., relative vorticity of the storm vortex, in the units of Cyclonic Vorticity Unit, CVU, 1 CVU=$1.0*10^{-5}$ s^{-1}) between ERA5 pre-TCG vortex and ERA5 TC vortex at the observed TCG time, for the same TCs, in the globe over 1980–2018. Red dot is for the average difference in the time and intensity (-4.6 days, -2.7 CVU) of all TCs, and cross is for standard deviation of the difference in the time and intensity.

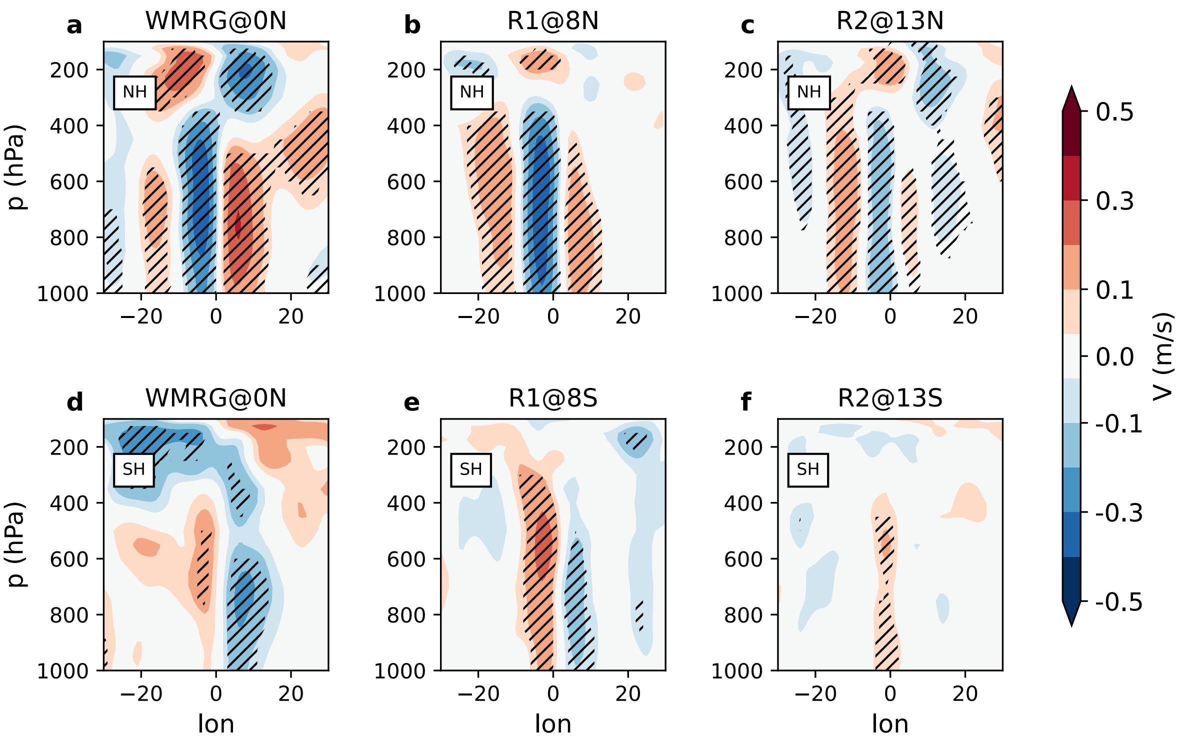

Supplementary Figure 13: Vertical profile of equatorial waves composited onto all pre-tropical cyclogenesis (pre-TCG) events

(a) Composite means of vertical profile of Westward-moving Mixed Rossby-Gravity wave (WMRG) meridional wind velocity (*V*) at the equator (0°N) onto all pre-TCG events, with respect to the vortex longitude and event time of pre-TCG, in the Northern Hemisphere (NH), over 1980–2018. (b-c) as (a), but for mode number 1 Rossby wave (R1) and mode number 2 Rossby wave (R2) meridional wind velocity at 8°N and 12°N, respectively. 0°N, 8°N and 13°N are chosen because WMRG, R1 and R2 waves have the maximum values of *V* at these three latitudes, respectively.

(d-f) as (a-c), but for the Southern Hemisphere (SH). The hatched area shows significant composite at the 95% confidence level.

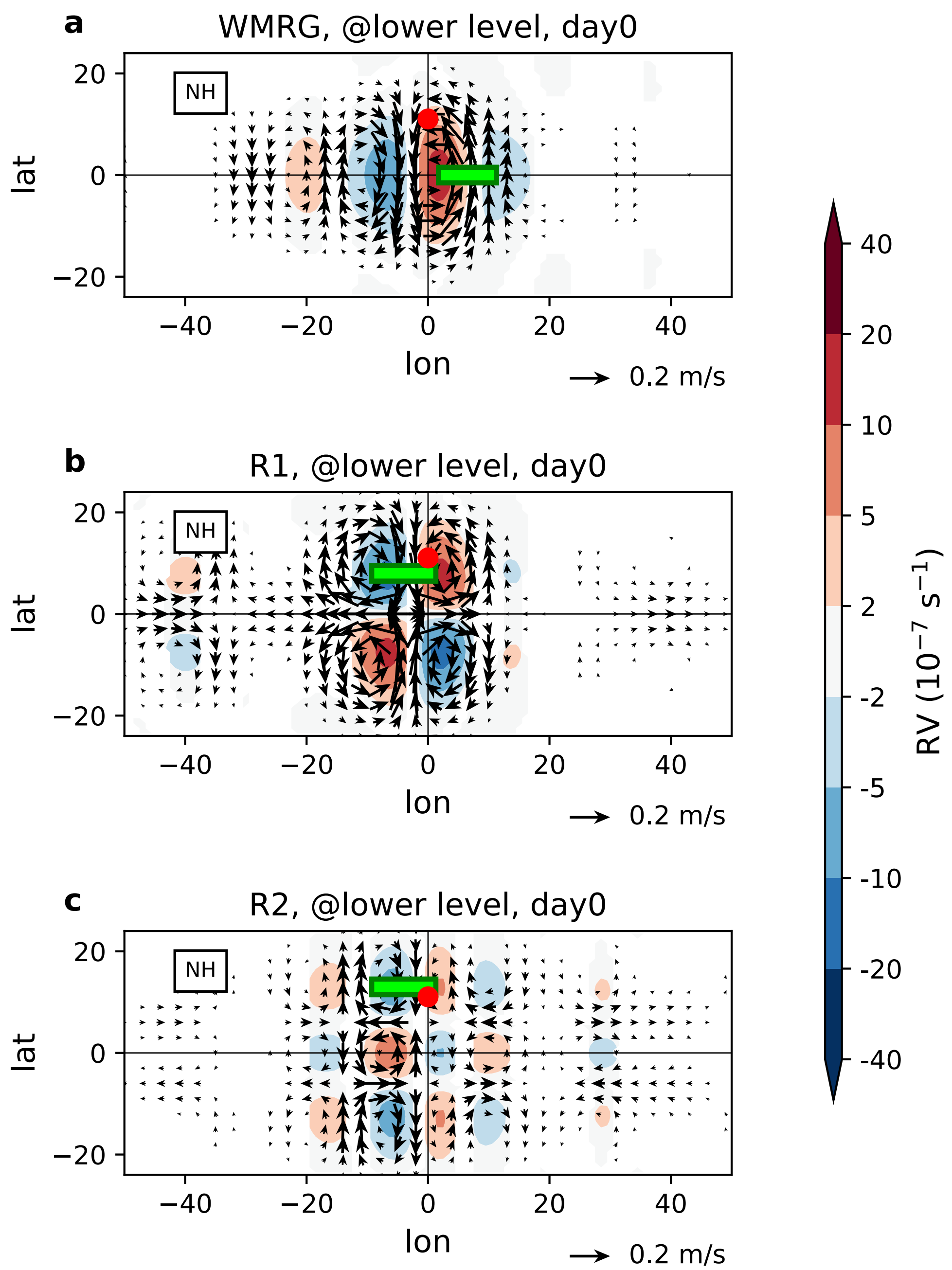

Supplementary Figure 14: The pre-tropical cyclogenesis (pre-TCG) and wave matching in the Northern Hemisphere (NH)

(a) Illustration of Westward-moving Mixed Rossby-Gravity wave (WMRG) horizontal winds (vectors) in the lower troposphere, with respect to the longitude and time of pre-TCG event, in the NH. (b-c) as (a), but for mode number 1 Rossby wave (R1) and mode number 2 Rossby wave (R2), respectively. Red dot shows the latitude of pre-TCG vortex; the green bar shows the relative location where the wave meridional wind is averaged in defining the phase matching of pre-TCG and waves. The latitude of the green bar is 0°N, 8°N and 13°N for WMRG, R1 and R2 waves, respectively, where the wave meridional wind has the maximum values. Shading shows relative vorticity (RV, in the units of 10^{-7} s^{-1}) of wave winds in the lower troposphere. The lower troposphere is averaged over 900, 850, 800, 750 and 700 hPa. For detailed criteria in the phase matching, see the Methods.
